# Eye movement desensitization and processing for adolescents with major depressive disorder: study protocol for a multi-site randomized controlled trial

**DOI:** 10.1186/s13063-023-07226-y

**Published:** 2023-03-20

**Authors:** C. C. Paauw, C. de Roos, M. G. T. Koornneef, B. M. Elzinga, T. M. Boorsma, M. A. Verheij, A. E. Dingemans

**Affiliations:** 1 Department Youth, GGZ Rivierduinen, Sandifortdreef 19, 2333 ZZ Leiden, the Netherlands; 2 Department of Child and Adolescent Psychiatry, Amsterdam University Medical Centre, Amsterdam, the Netherlands; 3 Institute of Psychology, Leiden University, Leiden, the Netherlands; 4 GGZ Delfland, Delft, the Netherlands; 5 GGZ Rivierduinen Eating Disorders Ursula, Leiden, the Netherlands

**Keywords:** EMDR, Adolescents, Major depressive disorder, Randomized controlled trial, Treatment, Trauma

## Abstract

**Background:**

Major depressive disorder (MDD) is one of the most common mental disorders in adolescence carrying a serious risk of adverse development later in life. Extant treatments are limited in effectiveness and have high drop-out and relapse rates. A body of literature has been published on the association between distressing/ traumatic experiences and development and maintenance of MDD, but the effectiveness of a trauma-focused treatment approach for MDD has hardly been studied. This study aims to determine the effectiveness of eye movement desensitization and reprocessing (EMDR) therapy as stand-alone intervention in adolescents diagnosed with MDD.

**Methods:**

This study will be a randomized controlled trial with two conditions: (1) EMDR treatment (6 sessions) and (2) waiting list condition (WL: 6 weeks, followed by EMDR treatment). First, participants receive a baseline measure after which they will be randomized. Participants will be assessed post-intervention after which the WL participants will also receive six EMDR sessions. Follow-up assessments will be conducted at 3 and 6 months follow-up. Study population: In total, 64 adolescents (aged 12–18) diagnosed with a major depressive disorder (DSM-5) and identified memories of at least one distressing or traumatic event related to the depressive symptomatology will be included. Main study parameters/endpoints: Primary outcome variables will be the percentage of patients meeting criteria for MDD classification, and level of depressive symptoms. Secondary outcome measures include symptoms of PTSD, anxiety, and general social-emotional problems. At baseline, family functioning and having experienced emotional abuse or neglect will be assessed to explore whether these factors predict post-treatment outcome.

**Discussion:**

With the present study, we aim to investigate whether EMDR as a trauma-focussed brief intervention may be effective for adolescents with a primary diagnosis of MDD. EMDR has been proven an effective treatment for traumatic memories in other disorders. It is hypothesized that traumatic memories play a role in the onset and maintenance of depressive disorders. Particularly in adolescence, early treatment of these traumatic memories is warranted to prevent a more chronic or recurrent course of the disorder.

**Trial registration:**

International Clinical Trial Registry Platform (ICTRP): NL9008 (30–10-2020).

## Administrative information

**Table:** 

Title {1}	Effectiveness of Eye Movement Desensitisation and Reprocessing (EMDR) for Adolescents with Major Depressive Disorder
Trial registration {2a and 2b}	International Clinical Trial Registry Platform (ICTRP) Registered on October 30, 2020 https://trialsearch.who.int/Trial2.aspx?TrialID=NL9008 Title Effectiveness of EMDR for adolescents with Major Depressive Disorder Scientific title Effectiveness of EMDR for adolescents with Major Depressive Disorder Study type Interventional Control group Waiting list Grouping Parallel Arms 2 or more arms, randomized Masking Single Target size 64 Inclusion criteria (a) age 12–18 years (b) Major Depressive Disorder (MDD) as primary classification (DSM-5) (c) identified memories of at least one distressing or traumatic event related to the depressive symptomatology Exclusion criteria (a) psychotic symptoms that require immediate treatment (b) suicidal attempt or serious non-suicidal self-injury requiring hospitalization in the past month (c) substance dependence (d) IQ estimated to be ≤ 80 based on information from the referral letter or diagnostic phase (e) insufficient Dutch language skills Start date 2020–11-02 Stop date 2023–04-30 Diseases Major Depressive Disorder Interventions Participants in the EMDR intervention group receive EMDR treatment during six weeks (six sessions). Participants in the waiting list condition receive EMDR treatment during six weeks after a waiting time of six weeks Primary outcome Main parameter will be the effect of EMDR treatment on depressive symptomatology. Depressive symptoms will be measured by the CDI-2 and K-SADS-PL-5 Secondary outcome Secondary parameters focus on the effect of EMDR treatment on comorbid PTSD, anxiety and overall social-emotional problems, measured by CATS, SCARED and SDQ. We will examine whether baseline posttraumatic stress symptoms severity, family functioning (measured by the FAD) and having experienced emotional abuse or neglect (CTQ) significantly predicts post-treatment outcome MEC approved Yes Multicenter Yes Randomized Yes Issueing body Medisch-ethische toetsingscommissie Leiden Den Haag Delft, the Netherlands Source ID NL77425.058.20 Funding sources Vereniging EMDR Nederland; EMDR Europe Association Old NTR ID N/A Date registered 2020–10-30
Protocol version {3}	October 3 2022 Version 2
Funding {4}	The present study is supported by grants from Vereniging EMDR Nederland and EMDR Europe Association
Author details {5a}	GGZ Rivierduinen, Department Youth, Leiden, the Netherlands C.C. Paauw (corresponding author) Department of Child and Adolescent Psychiatry, Amsterdam University Medical Centre, Amsterdam, The Netherlands C. de Roos GGZ Rivierduinen, Department Youth, Leiden, the Netherlands M.G.T. Koornneef Leiden University, Institute of Psychology, Leiden, the Netherlands B.M. Elzinga GGZ Delfland, Delft, the Netherlands T.M. Boorsma GGZ Rivierduinen, Department Youth, Leiden, the Netherlands M.A. Verheij GGZ Rivierduinen Eating Disorders Ursula, Leiden, the Netherlands A.E. Dingemans
Name and contact information for the trial sponsor {5b}	Trial sponsor: GGZ Rivierduinen, Leiden, The Netherlands Contact name: Caroline Weststrate, director Address: Sandifortdreef 19, 2333 ZZ Leiden, The Netherlands E: c.weststrate@rivierduinen.nl T:(+ 31)718,908,888
Role of sponsor {5c}	The funding sources had no role in the design of this study and will not have any role during its execution, analyses, interpretation of the data, or decision to submit results

## Introduction

### Background and rationale {6a}

Major depressive disorder (MDD) is one of the most common psychiatric disorders of adolescence [1]. It is estimated that 14 to 25% of adolescents experience at least one episode of a depressive disorder before reaching adulthood [2]. MDD is a debilitating disease, often resulting in impairment in functioning on multiple life domains [3, 4]. Furthermore, multiple studies associated MDD with adolescent onset with other mental health disorders, elevated suicidal risk and physical complaints later in adult life [4, 5] as well as with social and legal problems [6]. Current treatments show limited effectiveness and high drop-out and relapse rates. Cognitive behavioural therapy (CBT) is the recommended treatment for MDD in adolescents [7]. A meta-analysis [8] only showed a small effect sizes for psychological treatment of MDD (i.e. Cohen’s *d* = 0.29). These findings suggest that it is of paramount importance to develop innovative strategies for treatment of adolescent MDD.

It has long been recognized that distressing and traumatic experiences relate to the development and maintenance of MDD [9, 10]. More specifically, it was found that traumatic interpersonal experiences (such as humiliation and entrapment) [11], and different forms of childhood abuse, primarily emotional abuse and emotional neglect [10, 12], are related to MDD. Moreover, having a history of childhood trauma predicts poor efficacy of treatment [13–15]. This highlights the importance of identifying trauma histories and possibly adding trauma-focused interventions in treating depressed adolescents [14]. In addition, a developing body of research highlights the role of family relationships and interactions as being particularly relevant to the onset and maintenance of MDD in adolescents [16–18]. Family functioning predicts onset of adolescent MDD [19] and is rated as more dysfunctional in families with a depressed adolescent [20] compared to families without mental disorders. Furthermore, family dysfunctioning negatively affects treatment outcome [16, 21].

According to the recent treatment guidelines, EMDR therapy is one of the recommended treatments for post-traumatic stress disorder (PTSD) [22, 23]. EMDR therapy has proved effective in processing memories of distressing events [24]. Preliminary evidence for the efficacy of EMDR therapy in the treatment of MDD in adults has been demonstrated in multiple case studies [25, 26] and randomized controlled trials. Studies investigating EMDR therapy as an adjacent therapy to CBT [27], to pharmacological treatment [28, 29] and to inpatient treatment [30, 31] obtained promising results. As a stand-alone treatment, EMDR was proven effective in reducing depressive symptoms [32], even for patients with long-term [33] or treatment-resistant depressive disorder [29]. Moreover, significant decreases of trauma [32] and anxiety symptoms [29] were obtained with EMDR treatment of MDD, as well as increases of social functioning [29] and quality of life [32].

Findings from a recent meta-analysis [34] suggest that EMDR can be an effective treatment for depression. Moreover, studies using EMDR showed not only a large effect, but results of EMDR treatment were superior to non-trauma-focused CBT. However, these studies included adult patients only. Our group conducted one of the first studies investigating the effectiveness EMDR therapy in a group of 32 adolescents with a primary diagnosis of MDD [35]. After six EMDR sessions, 60.9% of the adolescents who finished treatment no longer met DSM-IV criteria of MDD. In addition, comorbid anxiety, post-traumatic stress symptoms and somatic complaints were significantly reduced. At three-month follow-up, these results were maintained. Since this pilot study only included a small sample size and no control group, higher-quality studies are needed to explore these promising results.

### Objectives {7}

Building on the study by Paauw et al. [35], the current study aims to investigate whether EMDR therapy is more effective in comparison to a waiting list control condition.

#### Hypothesis 1

EMDR therapy is associated with a significant decrease in severity of depressive symptoms and decrease in percentage of patients meeting DSM-5 criteria for MDD compared to the waiting list.

Outcome measures: Total score on the CDI-2 and the percentage of patients meeting DSM-5 criteria for MDD (K-SADS-PL-5).

#### Hypothesis 2

After treatment with EMDR, participants report less comorbid PTSD, anxiety and social/emotional symptomatology in comparison to those in the waiting list control condition.

Outcome measures: severity of PTSD (CATS), anxiety (SCARED) and social/emotional symptoms (SDQ).

The second aim of this study is to investigate predictors of treatment outcome. We will test the following predictors: baseline PTSD symptoms (CATS), family functioning (FAD) and experienced emotional abuse or emotional neglect in childhood (CTQ).

#### Hypothesis 3

A higher level of post-traumatic stress symptoms at baseline predicts a stronger post-treatment reduction in depressive symptoms.

Outcome measures: reduction of depressive symptoms (CDI-2 total score), severity of post-traumatic stress symptoms (CATS total score).

#### Hypothesis 4

A higher level of family dysfunction at baseline predicts smaller post-treatment reductions of depressive symptomatology.

Outcome measures: severity of depressive symptoms (CDI-2 total score), family functioning (FAD, general functioning score and dimension scores).

#### Hypothesis 5

A higher level of emotional abuse or neglect at baseline predicts smaller post-treatment reductions of depressive symptomatology.

Outcome measures: reduction of depressive symptoms (CDI-2 total score), experience of emotional abuse or neglect (respective subscales CTQ).

### Trial design {8}

**Fig. 1:**
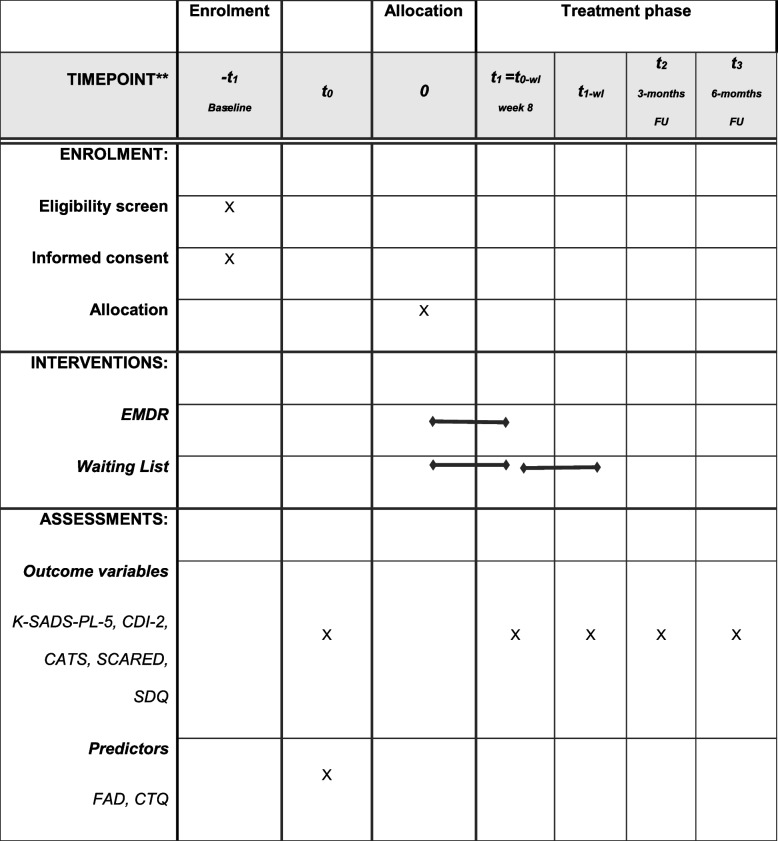
Participant timeline. *Recommended content can be displayed using various schematic formats. See SPIRIT 2013 Explanation and Elaboration for examples from protocols. **List specific time points in this row

This study is designed as a randomized, controlled, single-blinded two-center superiority trial with two parallel groups. All patients meeting the inclusion criteria are asked for written informed consent to participate in the intervention study. For consenting participants, baseline measures (T0) are completed subsequently. Randomization will be performed as block randomization with a 1:1 allocation stratified per treatment location. An independent researcher will conduct randomized allocation by using the SPSS function to produce random numbers. Hence, the main researcher will be blind to the randomization process. Randomization will take place in blocks of 10 participants. After completing the baseline assessments (T0), participants will be informed in which group they have been randomized. Assessments (see Fig. 1) are scheduled pre-treatment (T0), post-treatment (T1), and at 3-month (T2) and at 6-month (T3) follow-up. Assessment will be done by a team of independent assessors (i.e. trained clinicians and supervised master level students) who are blind for condition. Participants in the waiting list condition are offered EMDR treatment after T1 and are subsequently assessed post-treatment and at 3 and 6 months follow-up (see Fig. 1). For participants in the waiting list condition, T1 will be labelled as T0WL when they enter treatment (*t*_*1*_ = *t*_*0-wl*_).

## Methods: participants, interventions and outcomes

### Study setting {9}

Participants will be recruited from outpatient clinics of GGZ Rivierduinen Department Youth (locations Leiden, Katwijk, Leidschendam and Zoetermeer) and GGZ Delfland (locations: Bergschenhoek, Delft, Naaldwijk, Nootdorp en Schiedam), two institutions for mental health care in the Netherlands.

### Eligibility criteria {10}

Inclusion criteria for participants:

- Age 12–18 years
- Major depressive disorder (MDD) as primary diagnosis (DSM-5)
- Identified memories of at least one distressing or traumatic event related to the depressive symptomatology

Exclusion criteria for participants:

- Psychotic symptoms that require immediate treatment
- Suicidal attempt or serious non-suicidal self-injury requiring hospitalization in the past month
- Substance dependence
- IQ estimated to be ≤ 80 based on information from the referral letter or diagnostic phase
- Insufficient Dutch language skills

### Who will take informed consent? {26a}

Prior to the study, potential participants and their parents will be approached by the researcher or the research assistant and receive oral and written information about the content, purpose and research design. If all parties agree, informed consent will be signed by both participants and their parents.

### Additional consent provisions for collection and use of participant data and biological specimens {26b}

On the consent form, participants will be asked if they agree to use of their data should they choose to withdraw from the trial. Participants will also be asked for permission for the research team to share relevant data with people from the Universities taking part in the research or from regulatory authorities, where relevant. This trial does not involve collecting biological specimens for storage.

## Interventions

### Explanation for the choice of comparators {6b}

Effectiveness of EMDR is compared to an inactive condition (waiting list). To our knowledge, this is the first study investigating the effectiveness of EMDR therapy on the reduction of adolescent depressive symptoms. The waiting list control group serves two purposes. First, it provides an untreated comparison for the EMDR group to determine if EMDR therapy is effective in this specific group. Second, it allows the wait-listed participants the opportunity to obtain the EMDR intervention after 6 weeks.

### Intervention description {11a}

Prior to participation, a patient safety plan will be made with each participant; this is part of the standard screening and assessment routine of the participating organizations.

#### EMDR therapy

The Dutch version of the standard EMDR protocol with age-specific adaptations for children and adolescents [36] will be used. This procedure includes eight phases: history taking, preparation, assessment, desensitization, installation, body scan, closure and re-evaluation. Before start of the treatment, a case conceptualization is made to guide the sessions. Memories are placed in a hierarchy based on the subjective units of disturbance (SUD), and will be treated subsequently from high to low SUD. If all depression-related target memories from the case conceptualization can be retrieved without emotional disturbance (i.e. SUD related to the memory is reduced to zero) in less than six sessions, a participant will be classified as an early completer.

Treatment consists of a maximum of six weekly 60-min individual treatment sessions. Each session will be followed by a 15-min meeting with the therapist, adolescent and one or both parents. The content of this meeting is discussed beforehand with the adolescent and comprises any one of the following elements: (1) a content outline of the session, (2) parents’ view on the course of symptoms in the week before the session and (3) the need and possibilities for emotional support of the adolescent after the session. During EMDR treatment, the number of adverse events such as symptoms exacerbation, crisis contacts and self-injurious or suicidal behaviour, will be recorded each session.

#### Waiting list

For participants in the waiting list condition, a therapist is available in case of crisis or sudden worsening of symptoms; a record will be made of such contacts.

### Criteria for discontinuing or modifying allocated interventions {11b}

Participants can leave the study at any time for any reason without any consequences. The investigator can decide to withdraw a participant from the study for urgent medical reasons.

### Strategies to improve adherence to interventions {11c}

For each participating location, 1–2 EMDR therapists will be recruited from the local staff. Therapists have at least completed the official Basic and Advanced EMDR training (approved by EMDR European Association (EMDREA). Group supervisions will be given monthly by a certified EMDR Europe Child and Adolescent Consultant (CdR). All EMDR sessions will be videotaped and all therapists participate in monthly 2-h supervisions by a certified EMDR Europe Child and Adolescent Consultant (CdR). Therapists will report their EMDR sessions through session reports that are shared with the consultant after each session. Upon request, additional supervision is provided via email and telephone.

### Relevant concomitant care permitted or prohibited during the trial {11d}

During participation, no concomitant care or interventions are permitted, except for stable doses of medication already prescribed before participation.

### Provisions for post-trial care {30}

After completing the EMDR intervention, the therapist evaluates the effects of treatment with the participant, his/her parents and the responsible practitioner. The therapist also consults the multidisciplinary staff to jointly decide if further treatment is indicated, and if so further treatment will be offered.

### Outcomes {12}

Table 1 presents an overview of assessment instruments used for each measurement time. Details of instruments are described below.

**Table 1.**
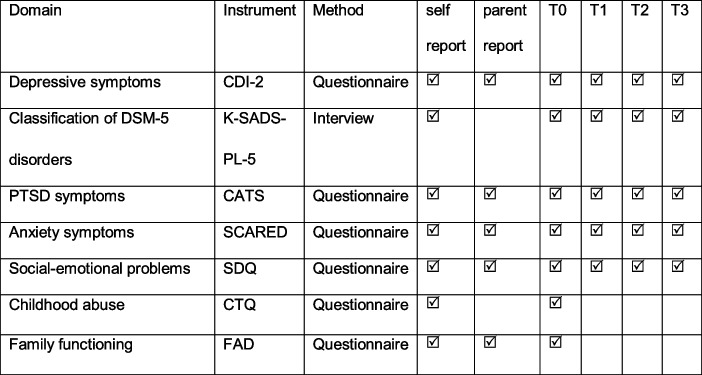
Assessment instruments

#### Primary outcome measures

##### **Depressivesymptoms**

The Dutch version [37] of the Children’s Depression Inventory-2 (CDI-2, Child and Parent Version) [38] will be used to assess affective, behavioural and cognitive aspects of depressive symptoms in the past 2 weeks. The CDI-2 includes 28 items dealing with sadness, self-blame, loss of appetite, insomnia, interpersonal relationships and school adjustment. The adolescent is asked to choose one of three sentences that best fits her/his feelings, thoughts and behaviours (e.g. “I am sad all the time”) in the past 2 weeks. These items are scored on a 3-point Likert scale (0–2, total range 0–56). The CDI-2 has good internal consistency and convergent validity [39]. For the Dutch version, internal consistency, test–retest reliability and convergent and divergent validity are high. For the parent version, internal consistency was high and convergent and divergent validity were moderate [37].

##### Structured interview for depression and comorbid disorders

The Schedule for Affective Disorders and Schizophrenia for School Age Children Present and Lifetime Version (K-SADS-PL-5) is a semi-structured diagnostic interview designed to assess current and lifetime diagnoses for affective, psychotic, anxiety, behavioural, substance abuse and eating disorders [40]. The clinician-based computer version of the K-SADS-PL-5 is used. For the purpose of the current study, questions about traumatic interpersonal experiences that have been identified as being connected to the onset of depressive episodes [25] (i.e. loss, separation and humiliation (being bullied/isolated/ignored), will be added to the K-SADS-PL-5 PTSD section.

The complete Youth Version is administered during pre-intervention assessment; at post-intervention and follow-up assessments, only the Depressive Disorders section, and the supplements for Suicidality and Sleep problems are administered.

All assessors are trained according to a programme consisting of observing live K-SADS COMP interviews and several supervised interviews. Each K-SADS COMP report will be reviewed and discussed by a supervisor. Moreover, all assessors attend 6-weekly intervision meetings.

#### Secondary outcome measures

##### Anxiety symptoms

The Dutch version of the revised Screen for Child Anxiety Related Emotional Disorders (SCARED-NL, Child and Parent Version) is used to assess signs of anxiety disorders in the past 3 months [41, 42]. The SCARED-NL is a 69-item inventory rated on a 3-point Likert-type scale (0 = ‘not true’ or ‘hardly ever true’; 1 = ‘somewhat true’ or ‘sometimes true’; 2 = ‘very true’ or ‘often true’; total range 0–82). Good psychometric properties (excellent internal consistency, large test–retest reliability and moderate to large parent–child agreement rates) of different versions of the SCARED were demonstrated in a large meta-analysis [43] and specifically for the Dutch version used in this study [42]. A 5-factor structure (panic/somatic, generalized anxiety, separation anxiety, social phobia, and school phobia) has been found repeatedly and has also been confirmed for the Dutch version. Since the reduced 5-item version of the SCARED has similar psychometrics to the full SCARED [44], the 5-item version will be used in this study.

##### Post-traumatic stress symptoms

The Dutch version of the Child and Adolescent Trauma Screen (CATS; Child and Parent Version) [45] explores individual trauma history using a checklist of 15 potentially traumatic events and frequency of each of 20 post traumatic stress symptoms, using a 4-point response scale ranging from 0 = never to 3 = almost always. The CATS has good to excellent reliability and sufficient convergent-discriminant validity for both self- and parent-report [45]. The psychometric qualities of the Dutch version are currently studied.

##### Global assessment of psychological problems

The Dutch adolescent version [46] of the Strengths and Difficulties Questionnaire (SDQ, Child and Parent Version) [47] is used as a global assessment of psychological problems. The SDQ consists of 25 items which are scored on a 3-point Likert scale ranging from ‘not true’, ‘somewhat true’ or ‘certainly true’ (total range 20–80). In this study’s analyses, the ‘total difficulties scale’ is used. The parent and self-report versions have an internal consistency that is generally acceptable in Dutch samples [46].

#### Predictors

##### Experiences of emotional neglect and emotional abuse

The Dutch version [48] of the Childhood Trauma Questionnaire (CTQ) [49] was adapted for adolescents and used to assess experiences of childhood maltreatment. The CTQ is a self-report list consisting of 28 items which are scored on a 5-point Likert scale. The CTQ has a good criterion validity in both a clinical and a healthy sample [50]. In the analyses, the subscales ‘Emotional neglect’ (range 5–25) and ‘Emotional abuse’ (range 5–25) are used. Internal consistency of these subscales were high in a Dutch sample [51].

##### Family functioning

The Dutch version [52] of the Family Assessment Device (FAD; Child and Parent Version) [53] is used to assess structural, organizational and transactional characteristics of families. The questionnaire covers 6 dimensions: affective involvement, affective responsiveness, behavioural control, communication, problem solving and roles. In addition, there is a 7th scale measuring general family functioning. Both dimension scores and the general functioning scale are used in the analyses. The FAD comprises 60 items rated on a 4-point scale. Scale scores are computed by adding the responses and dividing by the number of items in the scale with higher scores indicating worse levels of family functioning. The psychometric qualities of the English version have been studied extensively and were satisfactory to good [53, 54]. Results from studies on the Dutch version [55] were comparable.

### Participant timeline {13}

Figure 1 shows the participant timeline.

### Sample size {14}

A power analysis is calculated with G-Power. With the following assumptions: ANOVA with 2 groups and (EMDR and waiting list), 2 points of measurement (baseline and after treatment), the CDI-2 total score as outcome measure, effect size of F = 0.40, a power of 0.095 with *α* = 0.05, a total of 48 participants (24 participants per group) will be needed. In line with the drop-out rate (21.6%) we had in our first clinical study [35] and by those reported in previous intervention studies using EMDR with MDD patients (23% [26], 30% [29], 15% [30]), a maximum of 30% drop-out during treatment and follow-up trajectory is expected. Consequently, 64 adolescents will be included in the study, equally divided between EMDR and waiting list condition.

### Recruitment {15}

Adolescents will be referred by their general practitioner and receive a regular intake appointment. After this intake, a preliminary diagnosis is formulated and discussed in a multidisciplinary team. Further assessment (either psychological, psychiatric, family assessment or observational assessment at home or at school) is initiated on indication. At least, a clinician-based K-SADS-PL-5 interview will be conducted. In case both a major depressive disorder and memories of at least one distressing or traumatic event related to the depressive symptomatology are identified, the adolescents and their parents will be approached by the researcher or research assistant. They will explain the purpose of this study. If the adolescent (age 12–16 parents also need to sign) and the parents decide to participate, they will both sign the informed consent.

Expected recruitment will be 1–3 patients per month and expected duration of inclusion is 30 months.

## Assignment of interventions: allocation

### Sequence generation {16a}

Randomization will be performed as block randomization with a 1:1 allocation stratified per treatment location. An independent researcher will conduct randomized allocation by using the SPSS function to produce random numbers. To reduce predictability of a random sequence, details of any planned restriction (e.g. blocking) will be provided in a separate document that is only available to those who enrol participants and assign interventions. Hence, the researchers conducting the assessments will be blind to the randomization process. Randomization will take place in blocks of 10 participants.

### Concealment mechanism {16b}

Due to the nature of the trial, participants and therapists in the study cannot be blinded for condition. Assessors and therapists will emphasize the importance of blinding to the participants and remind them not to reveal the randomized treatment condition to the assessors. Therapists are supported by the principal investigator, who is not involved in the execution of the assessments. Assessors will be asked to report any case of unblinding, in which case, another assessor will repeat the entire measurement. The use of a validated password file which is not accessible for those involved in the assessment will ensure concealment until the randomization result is revealed.

### Implementation {16c}

An independent researcher from GGZ Rivierduinen will perform the randomization.

After randomization, the independent researcher will only notify the trial coordinator from the research team, who will inform the therapist and participant.

## Assignment of interventions: blinding

### Who will be blinded {17a}

Both assessors and data analysts will be blinded after assignment to interventions.

## Procedure for unblinding if needed {17b}

The design is open label with only outcome assessors and data analysts being blinded so unblinding will not occur.

## Data collection and management

### Plans for assessment and collection of outcomes {18a}

For the primary outcomes (depressive symptoms), different types of measurement—interview and questionnaires—are combined. Secondary outcomes are assessed by questionnaires. Both child and parent versions of the questionnaires will be used. A description of the study instruments can be found in the “Outcomes {12}” section.

The K-SADS interview will be administered via a secured video ZOOM-meeting, using the K-SADS computer tool with pre-programmed diagnostic algorithms based on DSM-5 criteria. The K-SADS interview will include demographic questions. Assessors will receive training in both the application and scoring of the K-SADS interview. All interview reports with allocated classifications are discussed with a supervisor.

All questionnaires will be administered via Qualtrics, an online survey system. Links to the questionnaires will be sent by email, including a unique participant number for every client. Participants and parents receive individual links. At T0, assessment is guided by an assessor via ZOOM video connection to enable participants to ask questions if they encounter difficulties or get support if they experience discomfort.

### Plans to promote participant retention and complete follow-up {18b}

The post-intervention (T1) is part of regular treatment evaluation and is planned in close cooperation with the EMDR therapist. Results of this measurement are handed over to the EMDR therapist, who will take the results into account to evaluate EDMR treatment with the participant, one of the parents and the overall responsible practitioner.

Appointments for follow-up assessments (K-SADS interview) will be made by telephone. One day in advance of the interview appointment, the link to the questionnaires is sent. Prior to the start of the K-SADS interview, the participant is either being thanked for filling in the questionnaires or kindly reminded to do so after the interview. If the participant does not respond within a week, a reminder is sent by email, and if necessary, followed by a phone call.

After the last measurement, participants receive 25 euros as compensation in the form of a gift voucher. To detect drop-out of specific groups, participants who discontinue the treatment programme will be asked to continue measurements.

### Data management {19}

Upon enrolment, each participant will be assigned a unique study ID. This is a substitute of their identity information and the only form of participant referral that will be presented on case report forms and that will be included in statistical analysis.

Under the supervision of the principal investigator, assessors fill in the case record forms and upload data to a secure server. Data are kept in a secured storage and only authorized staff are granted access by the principal investigator. Data completeness will be checked regularly by both our data managers and the independent researcher from GGZ Rivierduinen will regularly check the data files.

Participants can withdraw from the study at any moment without consequences. Data gathered from participants up until their withdrawal will still be used for analyses.

### Confidentiality {27}

The data of participants will be kept strictly confidential. Research data will be stored and managed by the research team. Names will be coded in unique study ID’s so that privacy will be guaranteed. Only researchers committed to this study have access to the coded data.

Non-coded data will be saved separately in locked filing cabinets. Only the research team, the accredited Medical Ethical Committee, and Inspectie Gezondheidszorg en Jeugd (IGJ) will have access to this data file. The confidentiality agreements will be stored in a secured folder on the network of GGZ Rivierduinen.

Data will be stored for a minimum of 15 years, according to guidelines from the Medical Ethical Committee.

### Plans for collection, laboratory evaluation and storage of biological specimens for genetic or molecular analysis in this trial/future use {33}

Not applicable, no samples collected.

## Statistical methods

### Statistical methods for primary and secondary outcomes {20a}

Data will first be summarized by frequency tables and descriptive statistics (mean, standard deviation, number and percentages of cases). To investigate potential group differences regarding demographic and clinical variables between the two conditions, *t*-tests and chi-square tests will be conducted. Summary tables will be provided for all variables at all assessment points. Intention-to-treat analyses will be conducted for all outcomes. Additionally, a completer analysis will be conducted.

#### Primary study parameter(s)

To investigate the effect of EMDR and waiting on the primary outcome (level of depressive symptoms, CDI-2) and secondary outcome variables (i.e. post-traumatic stress symptoms (CATS), anxiety symptoms (SCARED) and overall social-emotional problems (SDQ)), a linear mixed model analysis will be conducted. A chi-square test will be used to investigate whether there is a difference between conditions in the percentage of MDD diagnoses (K-SADS-PL; MDD, partial remission, full remission) at T1, comparing changes in the waiting list condition (T0–T1; *n* = 32) with changes in the EMDR condition (T0 (*n* = 32) + T0WL(*n* = 32) − T1).

Effect sizes will be calculated using Cohen’s *d.* Based on the mean difference between scores from baseline (T0) to post-treatment (T1) and from baseline (T0) to follow-up (T2, T3), the results will be divided by the pooled standard deviation (Cohen, 1988).

To investigate the predictive effect of baseline post-traumatic stress symptom severity (CATS), emotional abuse and emotional neglect (CTQ), and general family functioning (FAD) on treatment outcome, we will perform a linear mixed model analysis. All predictors will be entered separately and we will conduct Bonferroni post hoc corrections to correct for the number of analyses. The level of significance will be set at *α* = 0.05/4.

#### Secondary study parameter(s)

To investigate the effect of EMDR and waiting on secondary outcome variables (i.e. post-traumatic stress symptoms (CATS), anxiety symptoms (SCARED) and overall social-emotional problems (SDQ)), a mixed model analysis will be conducted. A chi-square test will be used to investigate whether there is a difference between the conditions in the percentage of MDD diagnosis (K-SADS-PL) at T1.

Effect sizes will be calculated using Cohen’s *d*. Based on the mean difference between scores from baseline (T0) to post-treatment (T1) and from baseline (T0) to follow-up (T2, T3), the results will be divided by the pooled standard deviation.

#### Other study parameters

For the prediction analyses, all treated participants, including participants from the former waiting list control group, will be combined. The four predictor variables (baseline post-traumatic stress symptom severity (CATS), emotional abuse, emotional neglect (CTQ) and general family functioning (FAD) scores will be entered separately in the linear mixed model analyses. Bonferroni post hoc corrections will be conducted in order to correct for the number of analyses. The level of significance will be set at *α* = 0.05/4.

### Interim analyses {21b}

Not applicable, there are no interim analyses.

### Methods for additional analyses (e.g. subgroup analyses) {20b}

Not applicable, there are no subgroups.

### Methods in analysis to handle protocol non-adherence and any statistical methods to handle missing data {20c}

In case of treatment drop-out, we will try to motivate participants to adhere to the scheduled measurements. If this fails, we will still use data gathered from participants up until their withdrawal for the analyses. An intention-to-treat method will be performed.

Due to the randomized design, multivariable models will not be used to adjust for confounding, and therefore, the absence of covariates will not affect the primary analysis.

### Plans to give access to the full protocol, participant-level data and statistical code {31c}

To guarantee participant anonymity, the data set on participant level will not be publicly available. The data and statistical code supporting the findings of the final study report will be available on reasonable request.

## Oversight and monitoring

### Composition of the coordinating centre and trial steering committee {5d}

The research teams consist of the principal investigator (CP), a clinical psychologist in training (MK), two senior researchers (CdR, AD), a professor of an affiliated university (BE) and a research assistant (MV). The principal investigator is responsible for daily monitoring, for contact with the independent researcher performing the randomization, and for all contacts with participating practitioners. Assessments will be coordinated by the second investigator (MK) and the research assistant to guarantee blindness to treatment condition. The research team bimonthly evaluates the progress of the study. The local principal investigator of participating institution GGZ Delfland (MB) was involved in the design of the study and will be involved in the practical implementation of the study and in the reporting of the results.

For participants, an independent expert is available for questions about the trial.

### Composition of the data monitoring committee, its role and reporting structure {21a}

Collection, storage and processing of the data will be performed by the research team, with the assistance of a research assistant and master degree students. Because of the low risks involved in this study, no official data monitoring committee will be formed. To promote data quality, regular data monitoring will be done by an independent peer auditor, not involved in the study. This auditor will randomly check the research file on inclusion pace, drop-outs and general presence of completeness of the data.

### Adverse event reporting and harms {22}

Adverse events are defined as any undesirable experiences occurring to a subject during the study, whether or not considered related to the intervention. During EMDR treatment, the number of adverse events such as symptom exacerbation, crisis contacts and self-injurious or suicidal behaviour will be recorded each session. All by participants, assessors or practitioners reported or observed adverse events will be recorded in the sessions forms, which are stored on a secured storage.

A serious adverse event (SAE) is defined—conform the guidelines of the Dutch Central Committee on Research Involving Human Subjects (CCMO)—as any untoward medical occurrence or effect that results in death, is life threatening (at the time of the event), requires hospitalization or prolongation of existing inpatients’ hospitalization or results in persistent or significant disability or incapacity.

The study group will report all SAEs through the web portal ToetsingOnline to the accredited Medical Ethical Committee, without undue delay after obtaining knowledge of the events (a period of 8 days of first knowledge to complete the initial preliminary report for SAEs that result in death or are life threatening; a period of maximum 15 days for all other SAEs).

### Frequency and plans for auditing trial conduct {23}

The investigators will check the research file on a weekly basis; peer auditor will monitor the file once a year.

### Plans for communicating important protocol amendments to relevant parties (e.g. trial participants, ethical committees) {25}

The current study builds on a pilot study [35] and uses the same treatment protocol. Therefore, major protocol modifications during the period of data collection are not expected. If a substantial modification is necessary, it will be first proposed to the Medical Ethical Committee. Only after approval modifications will be implemented.

### Dissemination plans {31a}

Regardless of the direction or magnitude of effects, study results will be disseminated in scientific peer-reviewed journals and presentations at conferences. Via presentations, chapters in books and supervision, clinicians will be informed about the specific characteristics of both the case conceptualization and dynamics during EMDR treatment.

Participants will receive the final study report on request.

## Discussion

The present study is the first single-blinded randomized controlled trial with a 6-month follow-up period that investigates the effectiveness of EMDR therapy on depressive and comorbid symptoms in a sample of adolescents. We expect that EMDR therapy is associated with a significant reduction in depressive and comorbid symptoms. We consider this of great importance, given the limited effectiveness of current psychotherapies for adolescent MDD [8, 56].

The current study has several strengths. Firstly, it is a multicentre outpatient trial with a variety of MDD patients and a large number of EMDR therapists, enhancing the external validity of the study. Secondly, thorough assessment of psychopathology is offered using a standardized DSM-5-based clinical interview, by assessors blind to treatment condition. Thirdly, a shorter treatment length, compared to current NICE guidelines [4]. Fourthly, the study includes two follow-up measurements, documenting the longevity of the hypothesized effects. Fifthly, treatment protocol adherence by EMDR therapists will be evaluated utilizing video recordings. This may help therapists to stay focused and stick to the protocol. Eventually, it will be possible to determine to what extent the protocol was applied correctly.

With the present study, we aim to investigate whether EMDR as a trauma-focused brief intervention may be effective for adolescents with a primary diagnosis of MDD. It is hypothesized that traumatic memories play a role in the onset and maintenance of depressive disorders. Especially in adolescence, early treatment of these traumatic memories is warranted to prevent a more chronic or recurrent course of the disorder. For adults, there is some evidence that EMDR is effective in treating traumatic memories in MDD [34]. It is expected that EMDR will decrease depressive symptoms as well as comorbid post-traumatic stress, anxiety, somatic complaints and overall social emotional functioning in adolescent patients with a primary diagnosis of a depressive disorder who also experienced at least one distressing or traumatic event related to the depressive symptomatology.

## Trial status

Trial NL9008, version 1.

Inclusion started on 02–11-2020. We expect to finish inclusion in April 2023.

## Data Availability

The researchers of the research team have access to the dataset. Any data required to support the protocol can be supplied on request by other people from the research team and regulatory authorities that are mentioned in the information letter.

## Abbreviations

CATS: Child and Adolescent Trauma Screen
CBT: Cognitive behavioural therapy
CCMO: Centrale Commissie Mensgebonden Onderzoek (Dutch Central Committee on Research Involving Human Subjects)
CDI-2: Children’s Depression Inventory-2
CTQ: Childhood Trauma Questionnaire
DSM-IV: Diagnostic and Statistical Manual of Mental Disorders, fourth edition
DSM-5: Diagnostic and Statistical Manual of Mental Disorders, fifth edition
EMDR: Eye Movement Desensitization and Reprocessing
EMDREA: EMDR European Association
FAD: Family Assessment Device
IGT: Inspectie Gezondheidszorg en Jeugd (Health Care and Youth Care Inspectorate)
K-SADS(-PL-5): The Schedule for Affective Disorders and Schizophrenia for School Age Children Present and Lifetime Version
MDD: Major depressive disorder
SCARED: Screen for Child Anxiety Related Emotional Disorders
SDQ: Strengths and Difficulties Questionnaire
SUD: Subjective Unit of Disturbance
WL: Waiting list
